# Enhancing Anti-Cancer Immune Response by Acidosis-sensitive Nanobody Display

**DOI:** 10.21203/rs.3.rs-4750804/v1

**Published:** 2024-08-16

**Authors:** Leah E. Knepper, Emily T. Ankrom, Damien Thévenin

## Abstract

One of the main challenges with many cancer immuno-therapies is that they depend on biomarkers for targeting. These biomarkers are often associated with tumors but are not specific to a particular tumor, which can lead to damage in healthy tissues, resistance to treatment, and the need for customization for different types of cancer due to the variations in targets. A promising alternative approach is to target the acidic microenvironment found in most solid tumor types. This can be achieved using the pH (Low) Insertion Peptide (pHLIP), which inserts selectively into cell membranes in acidic conditions, sparing healthy tissues. pHLIP has shown potential for imaging, drug delivery, and surface display. For instance, we previously used pHLIP to display epitopes on the surfaces of cancer cells, enabling antibody-mediated immune cell recruitment and selective killing of cancer cells. In this study, we further this concept by directly fusing an anti-CD16 nanobody, which activates Natural Killer (NK) cells, to pHLIP, eliminating the need for antibody recruitment. Our results demonstrate pH-sensitive insertion into cancer cells, activation of the CD16 receptor on effector cells, and successful targeting and destruction of cancer cells by high-affinity CD16 ^+^ NK cells in two cancer cell lines.

## Introduction

Cancer is a significant global health challenge characterized by uncontrolled cell growth and the ability to evade the immune system. Immunotherapy has emerged as a promising approach to combat cancer by utilizing the body's immune system. However, targeting cancer cells while sparing normal cells poses challenges that are further complicated by the heterogeneous nature of tumor microenvironments.

One notable challenge in current cancer immunotherapies is that they target cell surface self-antigens that are associated but not specific to tumor cells, resulting in damage to healthy tissues. For instance, FDA-approved antibody-drug conjugates like Brentuximab vedotin for Hodgkin's lymphoma and Trastuzumab emtansine for HER2-positive metastatic breast cancer target self-antigens that are also present on normal cell surfaces.[1] Relying on biomarkers for targeting can also lead to the survival and proliferation of cancer cells that have developed resistance to treatment. Therefore, it is essential to carefully consider these factors when developing effective and safe cancer therapies.[2–5]

On the other hand, the microenvironment surrounding tumor masses is typically acidic (as low as 6.0),[3–7] providing a potential universal targeting strategy for tumor masses. pH(Low) Insertion Peptides (pHLIPs) represent a new class of peptides with well-established tumor-targeting properties in animals and promising therapeutic applications.[5,8–22] For example, a pHLIP conjugate with the imaging agent indocyanine green is undergoing Phase I/II clinical trials.[23] pHLIP peptides can insert into cell membranes in the mildly acidic milieu typical of tumor sites, allowing for targeted delivery of therapeutic agents directly to tumor sites. Unlike cell-penetrating peptides, pHLIP's insertion does not disrupt the cell membrane or promote the formation of pores within the membrane.[24,25] They also insert into the cell membrane unidirectionally (presenting its N-terminus externally), enabling the display of diverse molecules on the cancer cell surface.[3–5,7,26,27] Therefore, pHLIP's targeting properties hold significant promise for enhancing cancer immunotherapy.

We previously used pHLIP to graft epitopes (e.g., FITC, dinitrophenol, and peptides) on the surface of cancer cells to recruit antibodies and activate effector cells, leading to selective cancer cell killing.[4,7] Building on this previous work and to eliminate the need for antibodies to bridge cancer and immune cells, this study aims to develop a new method for cancer immunotherapy, using pHLIP to display a nanobody selectively on the surface of cancer cells. This nanobody would activate CD16 receptors present on immune cells,[28] eliminating the need for antibody recruitment (Fig. 1). Nanobodies (single-domain antibodies or $V_H$H) offer several advantages for engineering proteins for immunotherapy, including their small size(~ 15k Da), better tissue penetration, ease of modification, high yield expression in various organisms,[29–32] easily folding,[31] stability,[31,33] and potentially low antigenicity in humans.[5,30] The CD16 (FCγRIII) receptor, expressed on NK cells, monocytes, and macrophages, is instrumental in mediating the immune response against neoplastic cells. Upon activation, this receptor triggers NK cell degranulation, culminating in the release of cytolytic agents like perforins and granzymes, which are crucial for the lysis of target cancer cells.[34–40] Combining an anti-CD16 nanobody and pHLIP (VHH-pHLIP) is expected to enhance the immune system's ability to recognize and destroy cancer cells, potentially offering a breakthrough in cancer treatment. Our approach is distinct from other nanobody display approaches in that immune cell recruitment is mediated by tumor acidity and not by recognizing an epitope at the surface of cancer cells. Our results show that selectively decorating the surface of cancer cells with an anti-CD16 nanobody using pHLIP recruits and activates NK cells, leading to targeted cancer lysis and death.

### Results and Discussion

## Design, Expression, and Purification of the $V_H$H-pHLIP Fusion

While other anti-CD16 nanobodies exist,[41,42] we chose to include in our design the C21 sequence identified by Behar et al.[28] because of its (1) small size, (2) available sequence, (3) stability and ability to refold readily, (4) high binding affinity to FCγRIII (10 nM), (5) ability to activate NK cells, and (6) established *in vitro* and *in vivo* potent anti-cancer activity.[43] Because pHLIP insertion is unidirectional (i.e., N-terminus oriented toward the extracellular environment), $V_H$H-pHLIP was constructed by fusing the sequence of C21 to the N-terminus of pHLIP through a Glycine-Serine flexible linker. A His-tag was added to the N-terminal to facilitate Ni-NTA purification (Fig. 1a **and Figure S1a**). Positioning the $V_H$H at the N-terminus ensures its display on cancer cell surfaces, rendering it readily accessible to engage with and activate CD16 receptors on immune effector cells. Briefly, the $V_H$H-pHLIP fusion and $V_H$H alone were expressed, purified, and refolded from *E. coli* inclusion bodies using Ni-NTA affinity chromatography following established methods for $V_HH$.[44] Identity and purity (93%) were determined by SDS-PAGE followed by coomassie staining (**Figure S2a, b**).

## Biophysical Characterization

Proper refolding was first assessed by circular dichroism (CD), which provides information on the secondary structure content of proteins. The CD spectrum of $V_HH$ alone in solution (**Figure S3a**) exhibits a negative peak in the 210–225 nm range, characteristic of the β-sheet structure predominantly found in immunoglobulins.[45,46] $V_HH$-pHLIP displays a similar CD spectrum in solution (**Figure S3b**). However, when large unilamellar vesicles (an established membrane mimic environment used with pHLIP [47,48]) are added, and the pH is decreased to 6.0, it transitions to a more α-helical structure, as indicated by the appearance of a second negative peak. We attribute this pH-mediated change to the characteristic folding pHLIP as an α-helix upon acidification. Additionally, a blue shift in tryptophan (Trp) fluorescence emission is observed when the pH is decreased, characteristic of the transition of Trp into a more hydrophobic environment and of the insertion of pHLIP into membranes (pHLIP has two tryptophan residues in its transmembrane region; **Figure S1a**). As expected, these changes are not as drastic as those observed with pHLIP alone because the overall β structure of VHH and its four tryptophan residues (Figure S1a) partially 'mask' the spectral signatures characteristic of pHLIP. Nevertheless, these results indicate that VHH is properly folded and that conjugating it to pHLIP does not significantly affect pHLIP's pH-dependent properties.

### VH-pHLIP Displays pH-Dependent Insertion in Cancer Cell Membranes

Our next objective was to demonstrate that $V_HH$-pHLIP can insert into the membrane and present $V_HH$ on the target cell's surface. Cervical HeLa cancer cells were treated with $V_HH$-pHLIP at either neutral pH (7.4) or acidic pH (6.0) for only 10 minutes to mimic the lower pH of the tumor microenvironment, washed, and incubated with fluorescently labeled anti-His tag antibodies and analyzed by flow cytometry. Because of the unidirectional membrane insertion of pHLIP, a higher fluorescence intensity is expected for cells treated with $V_HH$-pHLIP at an acidic pH. The results show that treating cells with 10 μM of $V_HH$-pHLIP at pH 6.0 resulted in a 3-fold increase in fluorescence intensity over the pH 7.4 conditions, suggesting that the $V_HH$ is surface exposed and available to bind with the anti-His tag antibodies (Fig. 2a). Moreover, $V_HH$ alone does not appear to bind to the surface of cells at either pH condition, further indicating that $V_HH$ surface display can be attributed to the membrane insertion of pHLIP. Surface display was also investigated by confocal microscopy. Briefly, HeLa cells were grown on coverslips and treated as described above. Only cells treated with $V_HH$-pHLIP at pH 6.0 showed a noticeable increase in green fluorescence around the periphery of the cells (Fig. 2b), suggesting surface display of the $V_HH$. While $V_HH$-pHLIP shows some unspecific binding at pH 7.4, it remains minimal when the concentration increases. Together, these results demonstrate the pH-dependent surface display of the nanobody by pHLIP.

### Surface-Displayed VH on Cancer Cells Activates the CD16 Signaling

We next sought to show that cancer cells decorated with $V_H$H-pHLIP in a pH-dependent manner could activate CD16 receptors expressed at the surface of effector cells. HeLa cells were treated with $V_H$H-pHLIP as described above and incubated with Jurkat-Lucia™ NFAT-CD16 reporter cells for 6 hours. Cells treated with $V_H$H-pHLIP at pH 6.0 are expected to display higher levels of $V_H$H on their surfaces, leading to higher activation of CD16 and luminescence intensity than cells treated at pH 7.4. Figure 3 shows that treating cells with $V_H$H-pHLIP results in a concentration- and pH-dependent increase in CD16 activation, with a 3-fold increase observed at pH 6.0 over pH 7.4 when treating with 0.16 μM $V_H$H-pHLIP, indicating that the nanobody interacts productively with effector cells. Maximum activation for both pH conditions is reached at a $V_H$H-pHLIP concentration of 1.25 μM, eliminating pH selectivity. The level of pH selectivity and lower (but concentration-dependent) unspecific binding at normal pH agree with the surface display results (Fig. 2), albeit activation occurs at much lower concentrations than observed for antibody recruitment. We hypothesize that these concentration differences may be due to the stronger affinity and stability of the interaction between the CD16 receptors for the $V_H$H compared to the affinity of the anti-6x His tag antibody for the His-tag. Moreover, using engineered Jurkat reporter cells is expected to be more sensitive because of their high level of CD16 expression and optimized signaling pathway.

These observations suggest that lower treatment concentrations, while possibly displaying less $V_H$H on the cell surface, may provide better selectivity than higher concentrations. Nevertheless, these results further confirm that the anti-CD16 nanobody is displayed at the surface of target cancer cells in a pH-dependent manner and accessible to interact with effector cells.

## $V_H$H-pHLIP Induces Cytotoxicity upon Natural Killer Cell Activation

We wanted to determine whether treatment with $V_H$H-pHLIP could induce the killing of target cancer cells through the pH-selective recruitment and activation of immortal NK-92 cells (haNK), expressing the F176V variant of the FCγRIIIA receptor (CD16), which confers a high affinity for the Fc domain of IgG.[4,49] We first determined the level of lactate dehydrogenase (LDH) release as a measure of membrane disruption (which happens quickly after exposure to NK cells) and overall cytotoxicity. Briefly, HeLa cells were treated with $V_H$H-pHLIP as described above and incubated with haNK cells for 4 hours. The level of LDH release was determined using the colorimetric CyQUANT LDH Cytotoxicity Assay (Invitrogen). As shown in Fig. 4a, pH-dependent target cell lysis was observed only when treated with 2.5 and 5 μM of $V_H$H-pHLIP, with a pH selectivity about 2-fold over physiological pH. The cytotoxicity effect is also concentration-dependent and appears to plateau at around 2.5 μM $V_H$H-pHLIP. On the other hand, cytotoxicity remains relatively low and constant at pH 7.4. It should be noted that adding haNK effectors inhibits the growth of target cells, even without any other treatments (Fig. 4a). Indeed, NK-92 cells exhibit non-specific killing activity against cancer cells.[49–52]

Finally, we determined the effect of treatment on cell viability using the MTT colorimetric assay. HeLa cells were treated with $V_H$H-pHLIP as described above and incubated with haNK cells for 48 hours. Since MTT measures cell viability, samples treated at pH 7.4 were expected to display higher percent cell viability than those treated at pH 6.0. Figure 4b shows a concentration- and pH-dependent cytotoxic effect, with an $IC_{50}$ of 1.1 μM for the pH 6.0 treatment, limited unspecific killing at pH 7.4, and a 2-fold difference in cell viability when compared to pH 7.4 treatment. Finally, lung cancer A549 cells also show a cytotoxic effect dependent on concentration and pH, although the effect is more limited (Fig. 4c). This result possibly exemplifies cancer cells' differential resistance to NK cells. For example, A459 has been shown to be highly resistant to NK cells.[53] Concentrations showing effectiveness are higher than observed with the activation of Jurkat cells (Fig. 3) because it may require more $V_H$H display to activate NK cells than engineered cells. Nevertheless, the $IC_{50}$ value determined by MTT is certainly the most relevant to the desired outcome: sustained suppression of cancer cell growth and division.

Altogether, our results show that treating target cancer cells with $V_H$H-pHLIP results in NK-mediated cell death in a pH-selective manner, providing a potential therapeutic window even for immune-resistant cancers.

### Conclusion

This work shows that cancer cells can be selectively labeled with a CD16-activating nanobody based on the acidity of the tumor microenvironment. By employing pHLIP for selective tumor localization and membrane anchoring, we have eliminated the need for a tumor-specific antigen, a major obstacle in current antibody-based therapies. Our study found that pH-mediated cancer cell decoration with $V_H$H led to CD16-mediated cell signaling in effector cells and NK-mediated cytotoxicity toward target cancer cells.

Our new approach has one main advantage over previous strategies that used antigen-pHLIP conjugates to recruit antibodies: it bridges the target cancer cells with the immune cells directly (immune synapse) without needing large antibodies (endogenous or exogenous).[54] Moreover, this approach enables the use of administered NK cells, which have emerged as a viable "off-the-shelf" alternative to T-cells, as they can be easily grown, are safe for allogeneic use, and have demonstrated superior safety in human clinical trials, even in high doses.[55–61] To that end, we project that $V_H$H-pHLIP will be administered and allowed to accumulate in the tumor until reaching an optimal tumor-to-background ratio, followed by the administration of NK cells. Additionally, the risk of toxicity to normal tissues can be reduced by limiting their in vivo expansion through irradiation before injection. Additionally, we do not expect $V_H$H-pHLIP conjugates to widely activate immune cells throughout the body and cause systemic toxicity while circulating because immune cell activation requires receptor and antigen clustering due to the membrane's two-dimensional surface, which is not possible in solution. Similarly, we anticipate our approach will not induce non-specific death of bystander immune cells. Even though pHLIP has been observed to integrate into macrophage membranes, there is no significant impact on other immune cells, such as T cells. Furthermore, activated macrophages prevent pHLIP from persisting on their surfaces due to their highly active membrane recycling, which transports pHLIP into endosomes.[21] Finally, although pHLIP displays non-specific binding and killing at pH 7.4 in cell culture, it is minimal and consistent with other studies findings.

Although this study demonstrates a new strategy for displaying nanobodies on the surface of cancer cells, in vivo studies are necessary to confirm its effectiveness further. We plan to enhance this proof-of-concept agent by investigating pHLIP sequences with improved targeting and pharmacodynamics capabilities (e.g., Var3 variant),[62] as well as recently developed anti-CD16 nanobodies with superior binding affinity, increased specificity (e.g., CD16 vs other Fc gamma receptors), and lowered antigenicity (i.e., by humanizing the sequence).[41,42,63] This work is currently in progress but extends beyond the scope of the present studies. Therefore, we anticipate that the newly developed agent, which combines pHLIP's established capabilities of targeting tumors and the immune-stimulating properties of the anti-CD16 nanobody, will prove to be an effective therapeutic solution.

### Materials and Methods

## Protein Expression and Purification of $V_H$H-pHLIP and $V_H$H

The DNA sequence coding for the $V_H$H-pHLIP fusion (**Figure S1a**) and $V_H$H alone (**Figure S1b**) were subcloned into a pET21b(+) vector (Biomatik). Plasmids were transformed into Rosetta (DE3) BL21 for over-expression. Colonies were selected from antibiotic-resistant plates and used to grow starter cultures in Lennox broth (LB); starter cultures were added into large volume (2 L) and grown at 37°C with shaking to an $OD_{600}$ of 0.6–0.7 and induced with 1 mM isopropyl β-D-1-thiogalactopyranoside (IPTG). Induced cultures were grown for an additional 4 h at 37°C with shaking and harvested by centrifugation at 10,000 rpm, 4°C for 15 min.

Cell pellets were resuspended in 50 mM $NaPO_4$, 300 mM NaCl (pH 8.0), and lysed by sonication at 4°C (output control: 2, duty cycle: 20%, 10 min). Samples were centrifuged at 13,000 rpm, 4°C for 10 min (subsequent centrifuge steps use the same parameters). Since $V_H$H-pHLIP and $V_H$H alone expressed as inclusion bodies, cell pellets were washed in 50 mM $NaPO_4$, 300 mM NaCl, 1% Triton, and 1 M urea (pH 8.0) and incubated on ice for 10 min before centrifugation. Two additional washes were done in the same buffer (no incubation) before washing twice with 50 mM $NaPO_4$, 300 mM NaCl (pH 8.0). Inclusion body pellets were solubilized in 50 mM $NaPO_4$, 300 mM NaCl, and 6 M urea (pH 8.0) under rotation overnight at 4°C and centrifuged at 12,000 rpm, 4°C for 25 min (removes insoluble debris) before 0.45 µm filtering (PVDF filter) and purifying by Ni-NTA affinity chromatography. HisPur™ Ni-NTA Resin (Thermo

Scientific) was incubated with solubilized inclusion bodies under rotation at 4°C for 2 h. The resin was washed with 50 mM $NaPO_4$, 300 mM NaCl, 6 M urea, and 10 mM imidazole (pH 8.0) before eluting with an increasing step gradient (100 mM, 200 mM, and so on, up to 500 mM) of imidazole. Elutions were pooled and concentrated using 10K MWCO Macrosep® Advance Centrifugal Device (Pall). Adding 50 mM $NaPO_4$, 300 mM NaCl, 6 M urea (pH 8.0) to the top of the centrifugal device served to remove imidazole by washing it into the flow through.

## Protein Refolding

Refolding conditions were based on those described by Bao, *et al*.[64] The $V_H$H was refolded from 6 M urea using a 1/10 dilution method. The refolding buffer contained 50 mM $NaPO_4$, 300 mM NaCl, 352 mM L-Arg (aggregation inhibitor), 1.05% (m/v) PEG-3350 (protein stabilizer), and 1.25 mM GSH (reduced glutathione) and 1 mM GSSG (oxidized glutathione), pH 8.0. Concentrated protein had β-mercaptoethanol (BME) added to a final concentration of 1 mM to reduce any mismatched disulfide bonds. Protein was added dropwise into the refolding buffer under stirring at 4°C and allowed to stir for a minimum of 16 h. Aggregates were pelleted by centrifugation at 12 000 rpm, 4°C for 10 min, and the supernatant was concentrated using 10K MWCO Pierce™ Protein Concentrator PES (Thermo Scientific). Concentrated protein was dialyzed into 40 mM $NaPO_4$, 200 mM NaCl (pH 8.0) in a 7K Slide-A-Lyzer® Dialysis Cassette, 0.5 mL – 3 mL capacity (Thermo Scientific). The final protein concentration was determined using the Micro BCA™ Protein Assay Kit (Thermo Scientific). Aliquots were flash-frozen in liquid nitrogen and stored at -70°C. When thawed for experiments, phenylmethylsulfonyl fluoride (PMSF; a serine protease inhibitor) was added to 1 mM final concentration.

## Sample Preparation for CD and Tryptophan Fluorescence Measurements

$V_H$H alone in 40 mM $NaPO_4$, 200 mM NaCl (pH 8.0) was diluted to a final concentration of 10 µM. 40 mM $NaPO_4$ (pH 8.0) was used to dilute the sample such that the final buffer composition was 40 mM $NaPO_4$, 100 mM NaCl (pH 8.0). $V_H$H-pHLIP in 40 mM $NaPO_4$, 100 mM NaCl (pH 8.0) was diluted such that the final concentration in each sample was 6 µM. Any necessary sample dilution was done with 5 mM $NaPO_4$ (pH 8.0). 1-palmitoyl-2-oleoyl-sn-glycero-3-phosphocholine (POPC) lipids were dried down and desiccated overnight under vacuum; lipids were rehydrated in 5 mM $NaPO_4$ (pH 8.0) for 30 min at 37°C with occasional mixing throughout. The resulting large multilamellar vesicles went through seven freeze/thaw cycles and were then extruded through a polycarbonate membrane with 200 nm pores using a Mini-Extruder (Avanti Polar Lipids) to yield large unilamellar vesicles (LUVs). $V_H$H-pHLIP was incubated with these LUVs at a 1:300 ratio for at least 5 min before adjusting the pH to the desired values with HCl, and samples were then incubated at RT for 30 min before analysis. Due to the inclusion of various buffers in samples, the final buffer composition was 27.3 mM $NaPO_4$ and 63.6 mM NaCl (at experimental pH).

## CD Spectroscopy

A Jasco J-815 CD spectrometer with a Peltier thermally controlled cuvette holder was used to record Far-ultraviolet (far-UV) CD spectra. Measurements were performed in a 0.1 cm quartz cuvette. CD intensities are expressed in mean residue molar ellipticity [θ] calculated from the following equation:

where $\theta_{obs}$ is the observed ellipticity in millidegrees, *l* is the optical path length in centimeters, *c* is the final molar concentration of the peptide, and *n* is the number of amino acid residues. CD spectra were acquired from 260 to 200 nm in 1 nm intervals at a 100 nm/min scan rate, and five scans were averaged for each sample. The spectrum of POPC liposomes was subtracted from all samples measured in the presence of liposomes.

## Tryptophan Fluorescence Spectroscopy

Fluorescence emission spectra were recorded using a Fluorolog-3 spectrofluorometer (HORIBA). The excitation wavelength was set at 280 nm, and the emission spectrum was measured from 300 to 450 nm. The excitation and emission slits widths were both 5 nm.

## Culture of Human Cancer Cell Lines and NK-92 Cells

Human cervical HeLa cancer and human lung cancer A549 cells were cultured in Cytiva HyClone™ Dulbecco's Modified Eagles Medium (DMEM) high glucose, with L-glutamine and sodium pyruvate, and supplemented with 10% fetal bovine serum (FBS), 100 units/mL penicillin, and 0.1 mg/mL streptomycin. Engineered Jurkat-Lucia™ NFAT-CD16 cells with the high-affinity CD16 allotype (V158) (InvivoGen) were cultured in Gibco™ Iscove's Modified Dulbecco's Medium (IMDM) high glucose, with glutamine, HEPES, and sodium pyruvate, and supplemented with 10% FBS, 100 units/mL penicillin, 0.1 mg/mL streptomycin, and 0.1 mg/mL Normocin™. After the second passage, media at every other passage contains 10 µg/mL blasticidin and 0.1 mg/mL Zeocin® to maintain selective pressure. Engineered $CD16^{+}$ NK-92 cells (ATCC) were cultured in Gibco™ Minimum Essential Medium Alpha (MEMα) no nucleosides, low glucose, with glutamine and sodium pyruvate, and supplemented with 0.1 mM BME, 0.2 mM inositol, 0.02 mM folic acid, 12.5% FBS, 12.5% horse serum, 200 units/mL interleukin-2 (IL-2), 100 units/mL penicillin, and 0.1 mg/mL streptomycin. Cells were cultured in a humidified atmosphere with 5% $CO_2$ at 37°C.

## Antibody Recruitment Assay

Dilutions of $V_H$H-pHLIP were made in phosphate-buffered saline (PBS, pH 7.4), and consistency in salt concentrations was maintained by adding 40 mM $NaPO_4$, 200 mM NaCl (pH 8.0). HeLa cells were harvested and washed once with PBS (pH 7.4). 400,000 cells were resuspended in PBS (pH 7.4) and treated in suspension with $V_H$H-pHLIP (various dilutions) such that the final concentrations were 10 µM, 7.5 µM, 5 µM, or 2.5 µM. Samples were incubated for 5 min at 37°C and then treated at either pH 7.4 or 6.0 for 10 min at 37°C. Cells were washed twice with cold PBS (same pH as treatment) and blocked in PBS with 3% BSA (pH 7.4) for 10 min on ice. A 1:1000 dilution of DyLight® 488 Anti-6x His tag antibody (Abcam, ab117512) in PBS with 3% BSA (pH 7.4) was incubated with the cells for 30 min on ice. Cells were washed once with cold PBS (same pH as treatment) and fixed in 4% PFA for 10 min on ice. The cells were then resuspended in PBS (same pH as treatment) and analyzed by flow cytometry using a BD

FACSCanto II flow cytometer with a 488 nm argon laser and a 530 nm/30 nm bandpass filter. Antibody recruitment with $V_H$H alone as a negative-control experiment was done using an identical procedure, simply substituting the $V_H$H alone for $V_H$H-pHLIP.

## Immunofluorescence Microscopy

Glass coverslips were treated with poly-L lysine for 2 h at 37°C and washed with PBS (pH 7.4) before adding cells. HeLa cells were seeded on coverslips and allowed to adhere overnight. Cells were treated with 6 µM $V_H$H-pHLIP as described above. After treatment, coverslips were washed once with PBS with 10% FBS (same pH as treatment) before fixation with 4% PFA for 10 min at RT with rocking. Coverslips were blocked in PBS with 3% BSA (pH 7.4) for 30 min with rocking. A 1:1000 dilution of DyLight® 488 Anti-6X His tag® antibody in PBS with 3% BSA (pH 7.4) was incubated on the coverslips for 1 h at RT with rocking. Coverslips were then washed four times with PBS (pH 7.4). A 1 µg/mL solution of Hoechst 33342 (Invitrogen) in PBS (pH 7.4) was incubated on coverslips for 10 min at RT with rocking before washing twice more with PBS (pH 7.4). Coverslips were mounted on glass slides with Fluoromount (Sigma-Aldrich) and allowed to dry before sealing. Slides were imaged with a Zeiss LSM880 scanning confocal microscope with a 40x objective.

## CD16 Receptor Activation Assay

$V_H$H-pHLIP was diluted in serum-free treatment media (prepared from powdered DMEM), and consistency in salt concentrations was maintained by adding 40 mM $NaPO_4$, 200 mM NaCl (pH 8.0). HeLa cells were seeded in a 96-well cell culture plate, grown to confluency and treated with increasing concentrations of $V_H$H-pHLIP as described above. Cells were washed twice with cold serum-free treatment media (same pH as treatment). IMDM (without Normocin™, blasticidin, or Zeocin®) was added to each well. Jurkat-Lucia™ NFAT-CD16 cells were harvested by centrifugation and were resuspended in IMDM (without Normocin, blasticidin, or Zeocin), and 200,000 cells were added to each well for a 5:1 effector-to-target cells ratio. The plate was centrifuged at 300 rpm for 1 min before incubating for 6 h at 37°C. The plate was gently shaken before transferring the media to an opaque white 96-well plate for luminescence measurements. QUANTI-Luc reagent (InvivoGen) was added to each well, and the luminescence was measured immediately with a 0.1 s reading time on an Infinite 200 PRO Plate Reader (Tecan).

## Cell Killing Assay Measured by Lactate dehydrogenase (LDH) Release

HeLa cells were seeded in a 96-well cell culture plate, grown to confluency, and treated with increasing concentrations of $V_H$H-pHLIP as described above. Cells were washed once with DMEM (no phenol red, 10% FBS, and pen-strep). Wells used for determining the maximum LDH activity and spontaneous LDH release were washed with the same media, and fresh media was added to them for the remainder of the experiment. $CD16^+$ NK-92 cells were harvested by centrifugation, resuspended in DMEM (no phenol red, 10% FBS, pen-strep), and 200,000 cells were added to each treated well for a 5:1 effector-to-target ratio.

The plate was centrifuged at 300 rpm for 1 min before incubating for 3 h 15 min at 37°C. Following the manufacturer's recommendations, LDH activity was measured using the colorimetric CyQUANT™ LDH Cytotoxicity Assay Kit (Invitrogen). 10X Lysis Buffer was added to wells for maximum LDH activity, and MilliQ water was added to wells for spontaneous LDH release. The plate was incubated for an additional 45 min at 37°C (total 4 h incubation). The plate was centrifuged at 2,500 rpm for 5 min before transferring the supernatant to a black clear-bottom 96-well plate. The Reaction Mixture solution (containing the substrate) was added to each well and incubated in the dark at room temperature for 40 min. Absorbance at 488 nm (10 nm bandwidth) and 690 nm (50 nm bandwidth) was measured on an Infinite 200 PRO Plate Reader (Tecan). Percent Cytotoxicity was calculated using the following equation:

$$\%\, Cytotoxicity = \frac{[Treated\ LDH\ activity] - [Spontaneous\ LDH\ release]}{[Maximum\ LDH\ activity] - [Spontaneous\ LDH\ release]} \times\ 100\%$$

Spontaneous LDH release and maximum LDH activity were measured after milliQ water and 10X Lysis Buffer were added, respectively.

## Cell Killing Assay Measured by MTT Assay

Target cells (HeLa, A549) were seeded in a 96-well cell culture plate (10,000 cells per well) the day before each assay and allowed to adhere overnight. The following day, cells were washed twice with serum-free treatment media (pH 7.4) and treated with increasing concentrations of $V_H$H-pHLIP as described above. Cells were washed three times with serum-free treatment media (pH 7.4) after the treatment. $CD16^+$ NK-92 cells were harvested by centrifugation and resuspended in serum-free treatment media. Effector cells were added to each well at a 2:1 effector-to-target ratio (20,000 cells per well), and the plate was centrifuged at 300 rpm for 1 min. The plate was incubated at 37°C for a 48 h recovery period. At the end of the recovery, the wells were washed twice with serum-free treatment media to remove the haNK cells; after washing, fresh serum-free media was replaced over the cells. Cell viability in each well was determined by incubating with MTT reagent prepared fresh as a stock at 5 mg/mL in PBS. 10 μL of the stock was added to each well, and the plate was incubated for 2 h at 37°C. All media was removed from the wells, and the resulting formazan crystals were solubilized in DMSO. DMSO was also added to three unused wells to provide a background absorbance reading. The absorbance of each well was measured at 580 nm (10 nm bandwidth) on an Infinite 200 PRO Plate Reader (Tecan). The background absorbance of DMSO (from triplicate wells of DMSO only) was subtracted from each sample before calculating viability.

$$\%\, Cell\, Viability = \frac{[Treated\ ABS_{580}]}{[No\, Treatment,\ pH\ 7.4\ ABS_{580}]} \times\ 100\%$$

### Abbreviations

BSA, bovine serum albumin; BME, β-mercaptoethanol; DMEM, Dulbecco’s Modified Eagles Medium; FBS, fetal bovine serum; IL-2, interleukin-2; IMDM, Iscove’s Modified Dulbecco’s Medium; LB, Lennox broth;

LDH, lactate dehydrogenase; MEMα, Minimum Essential Medium Alpha; MTT, 3-(4,5-Dimethylthiazol-2-yl)-2,5-diphenyltetrazolium bromide; NK, natural killer; PBS, phosphate-buffered saline; SDS-PAGE, sodium dodecyl-sulfate polyacrylamide gel electrophoresis.

### Declarations

## Conflict of Interest:

The authors declare no conflict of interest

## Author Contribution

L.K. and E.A. carried out the experiments. L.K and D.T. analyzed the data and wrote the manuscript with support from E.A.. D.T. conceived the original idea and supervised the project.

## Acknowledgments:

This work was supported by the National Cancer Institute grant R21CA267087 to DT.

## Data Availability:

The data supporting this study's findings are available from the corresponding author upon reasonable request.

## Figures

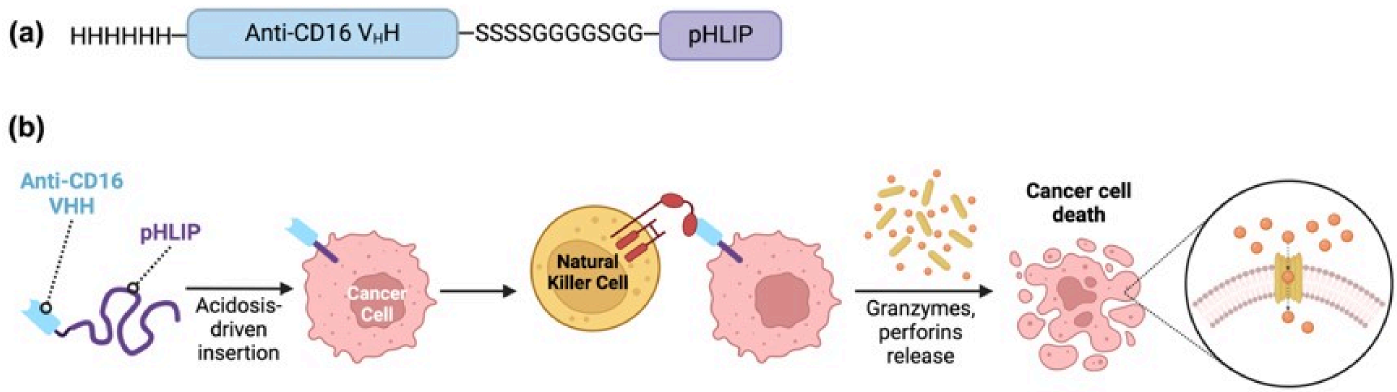

### Figure 1

Cartoon representations of **(a)** the anti-CD16 nanobody ($V_H H$) fusion with pHLIP and **(b)** the $V_H H$-pHLIP strategy for target cell killing developed in the present study (created with BioRender.com).

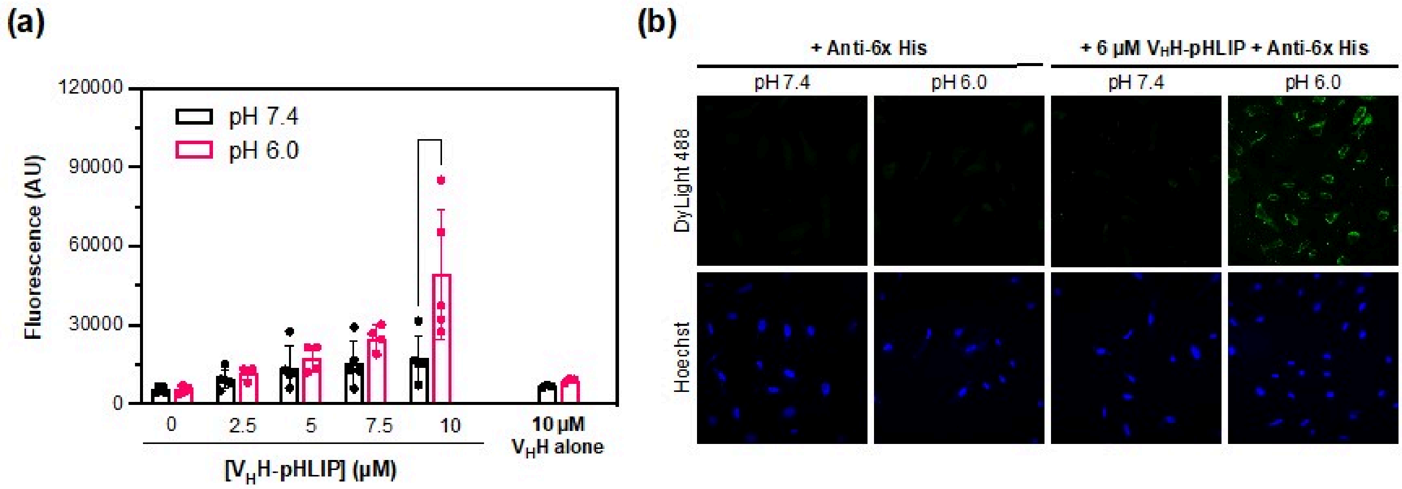

### Figure 2

pHLIP facilitates the display of anti-CD16 $V_H H$ to the surface of cancer cells. HeLa cells were treated with $V_H H$-pHLIP or $V_H H$ alone at pH 7.4 or 6.0 before washes and incubation with anti-His tag antibodies at pH 7.4. **(a)** Antibody recruitment was quantified by flow cytometry using DyLight 488 Anti-6x His tag antibody. Results are shown as mean ± SD (n=5). Statistical significance between samples was determined by t-test. **(b)** Representative immunofluorescence microscopy images of HeLa cells treated with $V_H$H-pHLIP at pH 7.4 or 6.0 with or without subsequence treatment with DyLight 488 Anti-6x His tag antibody.

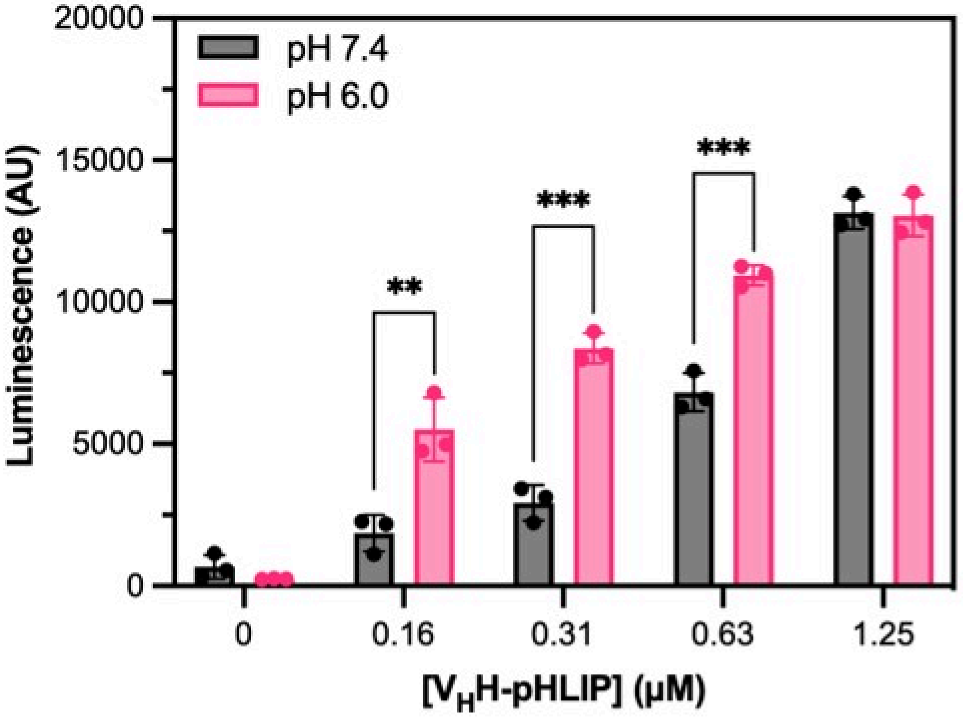

**Figure 3**

pHLIP-mediated surface display of $V_H$H on cancer cells activates CD16 receptors. HeLa cells were treated with $V_H$H-pHLIP at pH 7.4 or 6.0 and washed before incubating with Jurkat-Lucia NFAT-CD16 reporter cells 5:1 effector-to-target cells ratio. Activation of the reporter cells is seen by an increase in luminescent signal upon the addition of luciferase substrate. Statistical significance between pH treatments was determined by t-test. Error bars represent standard deviation (n = 3).

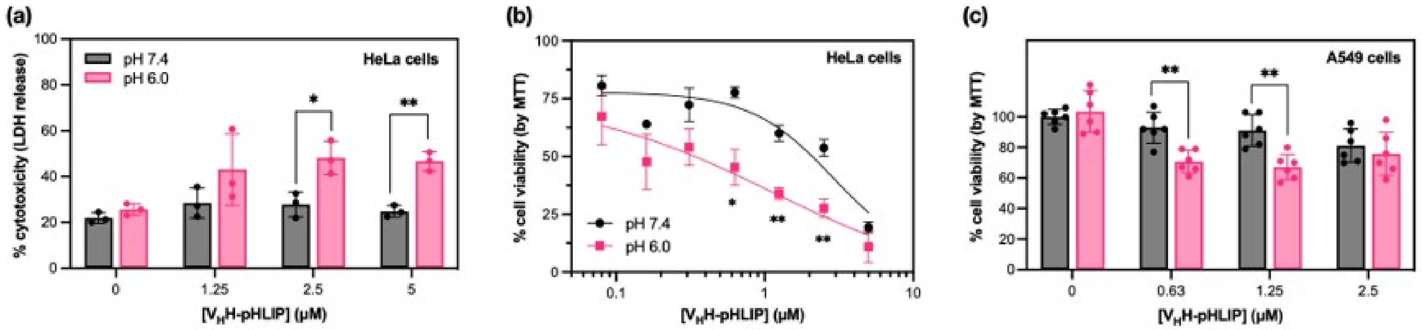

**Figure 4**

pHLIP-mediated $V_H$H cell surface display induces selective cancer cell killing by haNK cells. Cancer cells were incubated with increasing concentrations of $V_H$H-pHLIP for 10 min at pH 7.4 or 6.0 and further incubated with haNK cells. **(a)** Cytotoxicity towards HeLa cells was determined by LDH release after 4

hours of incubation with haNK cells at a 5:1 effector-to-target ratio. The effect of $V_H$H-pHLIP treatment and haNK cells on HeLa **(b)** and A549 **(c)** cells. Cell viability was measured using the MTT colorimetric assay after incubation with haNK cells for 48 h. Results are shown as mean ± SD (n=3-6), and the significance level between pH 7.4 and pH 6.0 treatment was determined by t-test.

## Supplementary Files

This is a list of supplementary files associated with this preprint. Click to download.

- KnepperSupplementaryFigures.pdf
